# Personalized neuron-specific enolase level based on EEG pattern for prediction of poor outcome after cardiac arrest

**DOI:** 10.1186/s13613-024-01406-y

**Published:** 2025-01-17

**Authors:** Juliette Pelle, Estelle Pruvost-Robieux, Florence Dumas, Antonin Ginguay, Julien Charpentier, Clara Vigneron, Frédéric Pène, Jean Paul Mira, Alain Cariou, Sarah Benghanem

**Affiliations:** 1 Medical Intensive Care Unit, AP-HP Centre Université Paris Cité, Cochin hospital, 27 rue du Faubourg Saint Jacques, Paris, 7501 France; 2 University Paris Cité – Medical School, Paris, France; 3 Neurophysiology and Epileptology Department, GHU Paris Psychiatry et Neurosciences, Sainte Anne Hospital, Paris, France; 4 INSERM, U1266, Pyschiatry and Neurosciences Institute (IPNP), Paris, France; 5 Emergency Department, AP-HP Paris Centre, Cochin hospital, Paris, France; 6 Clinical Chemistry Department, AP-HP Paris Centre, Cochin hospital, Paris, France

**Keywords:** Cardiac arrest, Prognostication, NSE, Electroencephalogram, Coma

## Abstract

**Background:**

After cardiac arrest (CA), the European recommendations suggest to use a neuron-specific enolase (NSE) level > 60 µg/L at 48–72 h to predict poor outcome. However, the prognostic performance of NSE can vary depending on electroencephalogram (EEG). The objective was to determine whether the NSE threshold which predicts poor outcome varies according to EEG patterns and the effect of electrographic seizures on NSE level.

**Methods:**

A retrospective study was conducted in a tertiary CA center, using a prospective registry of 155 adult patients comatose 72 h after CA. EEG patterns were classified according to the Westhall classification (benign, malignant or highly malignant). Neurological outcome was evaluated using the CPC scale at 3 months (CPC 3–5 defining a poor outcome).

**Results:**

Participants were 64 years old (IQR [53; 72,5]), and 74% were male. 83% were out-of-hospital CA and 48% were initial shockable rhythm. Electrographic seizures were observed in 5% and 8% of good and poor outcome patients, respectively (*p* = 0.50). NSE blood levels were significantly lower in the good outcome (median 20 µg/L IQR [15; 30]) compared to poor outcome group (median 110 µg/l IQR [49;308], *p* < 0,001). Benign EEG was associated with lower level of NSE compared to malignant and highly malignant patterns (*p* < 0.001). The NSE level was not significantly increased in patients with seizures as compared with malignant patterns (*p* = 0.15). In patients with a malignant EEG, a NSE > 45.2 µg/L was predictive of unfavorable outcome with 100% specificity and a higher sensitivity (70.8%) compared to the recommended NSE cut-off of 60 µg/l (Se = 66%). Combined to electrographic seizures, a NSE > 53.5 µg/L predicts poor outcome with 100% specificity and a higher sensitivity (77.7%) compared to the recommended cut-off (Se = 66.6%). Combined to a benign EEG, a NSE level > 78.2 µg/L was highly predictive of a poor outcome with a higher specificity (Sp = 100%) compared to the recommended cut-off (Sp = 94%).

**Conclusion:**

In comatose patients after AC, a personalized approach of NSE according to EEG pattern could improve the specificity and sensitivity of this biomarker for poor outcome prediction. Compared to others malignant EEG, no significant difference of NSE level was observed in case of electrographic seizures.

**Supplementary Information:**

The online version contains supplementary material available at 10.1186/s13613-024-01406-y.

## Introduction

Cardiac arrest (CA) remains a severe affection with more than 70% mortality after hospital admission. At admission, 80% patients present with a disorder of consciousness and 50% of survivors remains unconscious at day 3 [1]. Hypoxic Ischemic Brain Injury (HIBI) is the main prognostic factor, as two thirds of deaths are secondary to a withdrawal of life-sustaining therapies (WLST) related to a supposed pejorative neurological prognosis [2, 3]. Robust prognostication markers are thus necessary to avoid self-fulfilling prophecy. European recommendations highlight the need for a multimodal evaluation comprising clinical features (persistent disorder of consciousness without confounding factors, no pupillary and corneal reflexes at 72 h, status myoclonus in the first 72 h), electrophysiological features (bilaterally absent N20 on somato-sensory evoked potentials - SSEP, highly malignant electroencephalogram - EEG), imaging and biological markers of neuronal injury, that is plasma Neurone-Specific Enolase (NSE) level > 60 µg/L at 48 and/or 72 h. The presence of at least 2 markers among these 6 is highly predictive of poor neurological outcome [4].

EEG is useful to detect epileptic activity and evaluate the severity of HIBI [5]. According to the American Clinical Neurophysiology Society (ACNS) terminology and Westhall et al. classification [6], highly malignant patterns are predictive of a poor neurological outcome with 100% specificity. Conversely, the prognostic value of malignant patterns remain actually uncertain, with a false positive rate between 0 and 33% according to studies and patterns [5, 7]. Particularly, electrographic seizures or status epilepticus (SE) have uncertain prognostic value [8]. As such, anti-seizures medications are not systematically associated with neurological outcome improvement, seizures being interpreted then as a marker of extensive neuronal injury [9–13]. The serum NSE level reflects neuronal insult and is highly correlated with the degree of HIBI. Although recent recommendations have proposed the 60 µg/L cut-off as the best compromise between high specificity and acceptable sensitivity [4], different cut-offs were described from 33 to 120 µg/l in predicting poor neurological outcome [14–16]. Importantly, the prognostic value of EEG and NSE have mostly been studied independently, although the best NSE cut-off value could be personalized according to HIBI severity, notably reflected by EEG pattern [17, 18]. With a more specific approach according to EEG, we could improve the sensitivity of NSE level for the prediction of poor outcome.

Apart from CA patients, NSE is considered a robust marker of neuronal injury in SE, reflecting ongoing excitotoxicity and neuronal apoptosis [19, 20]. As such, electrographic seizures and SE could be considered as a confounding factor of NSE levels in CA patients.

We hypothesized that the blood NSE threshold for predicting an unfavorable outcome could be adjusted to the severity of HIBI reflected by EEG aspects. We also hypothesized that electrographic seizures may induce minor secondary brain damage and thus may not bias the analysis of the prognostic performance of NSE. Thus, the aim of the present study was to evaluate the optimal NSE threshold according to the EEG classification defined by Westhall et al., including electrographic seizures.

## Methods

### Population

We performed a retrospective analysis of a prospective registry at our tertiary cardiac arrest center. All consecutive adult patients were included if they were admitted in a comatose state as defined by a Glasgow Coma state (GCS) < 8 after an in- or out-hospital CA between February 2017 and December 2022. To be included, at least one EEG and one NSE level at 48 and/or 72 h after the CA should be performed. Early death (within 24 h of admission), brain death and CA of presumed neurological or traumatic origin were exclusion criteria. The patients’ relatives were informed that the data was collected for clinical research purposes.

### Data collection

This observational study followed the STROBE guidelines [21]. Demographic and clinical data were collected from medical files and local database. CA management data were collected using Utstein style, including initial cardiac rhythm and time to return of spontaneous circulation (ROSC). The in-hospital variables including causes of CA, ICU mortality, cause of death and WLST were also collected. Data collection and analysis were approved by the Ethics Committee of the French Society of Intensive Care (#CESRLF_12–384 and 20–41) and performed according to the French regulations on data protection (CNIL #MR004_2209691).

### EEG and NSE assessment

Video-EEG were recorded in the ICU by an experienced technician using a Deltamed Coherence device (Natus, Middelton, USA) or a Micromed Brain Quick device (Micromed SAS, Treviso, Italy). Recordings with 19 electrodes placed according to the 10–20 international system with additional ground and reference electrodes lasted 20 min. Repetitive auditive (calling of patient’s first name and family name, hand clapping), tactile and nociceptive (bilateral leg and arm touch and pinching) stimulations were conducted at least twice with a minimum of 10-seconds interval during the recording. The EEG recording was analyzed a second time for this study by two experienced neurophysiologists (SB, EPR), blinded to the others prognosis markers and to the patient’s outcome. Standardized terminology from the ACNS was used to describe the background activity, reactivity, and the presence of additional features such as rhythmic or periodic pattern, seizures and SE [22]. Each recording was then classified according to Westhall et al. into one of the mutually exclusive categories, namely highly malignant (suppressed or burst-suppression background, with or without superimposed periodic patterns), malignant (presence of at least one of the following: abundant rhythmic or periodic discharges, seizures or SE, discontinuous or low-voltage background, absence of reactivity) or benign (absence of malignant and highly malignant features) [6]. If patients had several EEG recordings, we used the first one for the analysis.

NSE was measured in serum samples. After collection in gel separator tubes, total blood samples were centrifuged within 4 h. Serum was stored at + 4–8 °C for analysis within 6 h. In case of delayed analysis (within 48–72 h), serum was stored at -40 °C. The NSE immunoassay was performed on Cobas^®^ analyzer (Roche Diagnostics, Meylan, France) using the commercial sandwich immunochemiluminescent Elecsys NSE assay (Roche Diagnostics, Meylan, France). The measuring range of NSE assay is 0.22–300 µg/L. Values above 300 µg/L were diluted manually 1:2 with the Diluent NSE, as recommended. The 95th percentile value is 16.3 µg/L (data from manufacturer). In our laboratory, within-run and between-run imprecision were < 1.0% and < 6.0% respectively, for concentrations of about 12 and 100 µg/L. Because of NSE overestimation, hemolyzed samples were not considered. NSE measurement was performed at 48 and 72 h (H48 and H72 respectively). For the present study, we primarily used the serum NSE concentration at H72, H48 data was included when H72 was not available.

### ICU management

Management of post-CA patients is standardized and includes targeted temperature management (TTM) at 33 °C (from 2017 to 2021) or 36 °C (from 2021 to 2022) using an external cooling device [23, 24], according to European guidelines [25]. TTM was initiated as soon as possible after admission and for 24 h in all patients that remain comatose after ROSC. Sedation protocol used a short-acting drugs (propofol and remifentanil) with protocolized adaptation according to the Richmond-Agitation-Sedation-Scale (RASS) (ESM1) [26–28]. Sedation was stopped as soon as spontaneous body temperature was restored. Management also comprised a multi-daily evaluation of consciousness using RASS as well as prevention of cerebral aggression of systemic origin. Awakening was defined by 3 consecutive RASS scores of -2 or more.

### Neurological prognostication process and withdrawal of life-sustaining therapies decisions

**Fig. 1:**
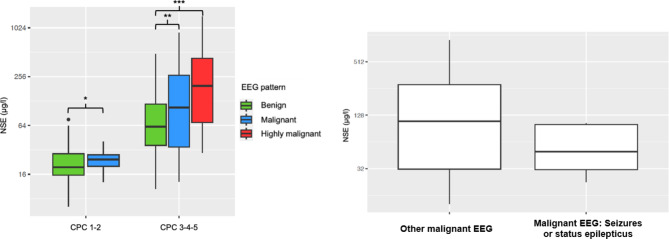
NSE distribution according to EEG patterns and neurological outcome at 3 months. NSE is illustrated in logarithmic scale. **A.** NSE according to EEG patterns and outcome. * *p* = 0.56; ** *p* = 0.18; *** *p* = 0.003; **B**. NSE according to the presence of epileptic features among malignant EEG patterns. We found no significant difference of NSE between seizures or status epilepticus and other malignant EEG pattern (*p* = 0.15). CPC: Cerebral Performance Category; NSE: neuro-specific enolase

In case of persisting coma 72 h after CA not explained by confounding factors, a multimodal prognostication protocol was used based on the 2015 and 2021 ERC-ESICM recommendations [4]. Before 2021, a poor neurological outcome was considered when patients presented a Glasgow motor score of 1 or 2 and at least one condition among: no pupillary and corneal reflexes, bilaterally absent N20 SSEP, refractory SE or suppression/burst-suppression EEG patterns [29]. After 2021, a poor neurological outcome was considered when at least two of the following conditions were observed: no pupillary and corneal reflexes after 72 h, bilaterally absent N20 SSEP, highly malignant EEG after 24 h, status myoclonus within 72 h after ROSC, diffuse and extensive anoxic injury on brain CT/MRI, NSE > 60 µg/l at 48 h and/or 72 h [4] (ESM2 - Fig.  1 supplementary material).

### Outcome assessment

The primary endpoint was neurological recovery according to the Cerebral Performance Category (CPC) scale at 3 months, evaluated by an independent trained researchers and assessed by telephone interview with the patient or their close relative (**ESM3**). If the information could not be obtained by telephone interview, we collected the neurological result from the medical records. A favorable neurological outcome was defined by a CPC level 1 or 2 (no, mild or moderate disabilities) (32).

### Statistical analysis

All data were collected on a single file. For descriptive analysis, continuous and discrete quantitative variables are described as mean (standard deviation) or median [interquartile range], and categorial variables are described as number and proportions of patients. Fisher exact test was used for qualitative variables and Mann-Whitney-Wilcoxon test for quantitative variables. We performed statistical analyses to produce ROC curves of the NSE predicting a CPC score of 3-4-5 at 3 months in the subgroups of interest. Optimal NSE thresholds were assessed according to each EEG pattern in predicting poor outcome with a 100% specificity and the highest sensitivity. Confidence intervals were defined as 95% and significance level was set at 0.05. Statistical analyses were performed using R version 4.2 (2022 The R Foundation for Statistical Computing).

## Results

Among the 606 patients included in our local registry between February 2017 and December 2022, 224 were not included mainly due to death in the first 24 h, leaving 382 patients potentially eligible. Among them, 227 patients were not included in the analysis because EEG and NSE were not performed or available (i.e., awakening before EEG or EEG raw data not available, NSE not performed or no NSE result available). Thus, 155 patients with at least one EEG and NSE performed and a 3 months follow-up were included (ESM4 - Fig. 2 supplementary material).

### Patients

Baseline characteristics are described in Table 1. Patients were 64 years old IQR [53; 72.5], and 74% were male. 83% were out-of-hospital CA and 48% were initial shockable rhythm. Overall ICU mortality was 71% with 91% of deaths attributed to WLST motivated by a presumed unfavorable neurological outcome. 39 patients (25%) had a favorable outcome, and 116 patients (75%) had an unfavorable outcome.

**Table 1:** Baseline characteristics of patients according to favorable and unfavorable outcome at 3 months

	Good outcome CPC 1–2 (*n* = 39)	Poor outcome CPC 3-4-5 (*n* = 116)	*p* value	
Age. median [IQR]	58 [50; 65.5]	66.5 [54.8; 75]	***0.005***	
Male. n (%)	30 (77%)	85 (73%)	*0.65*	
Out-of-hospital CA. n (%)	32 (82%)	96 (83%)	*1*	
Initial rhythm in VF/VT. n (%)	28 (72%)	47 (41%)	***0.001***	
Time to ROSC in minutes. Median [IQR]	18 [11; 24]	25 [21.8; 34]	***< 0.001***	
Cause of CA. n (%)				
Myocardial ischemia. n (%)	18 (46%)	28 (24%)	***< 0.001***	
Respiratory failure. n (%)	5 (13%)	58 (50%)	***< 0.001***	
Other cause. n (%)	16 (41%)	30 (26%)		
ICU Mortality. n (%)	110 (71%)*			
Cause of death. n (%)				
Multiple organ failure. n (%)	-	10 (9%)		
WLST. n (%)	-	100 (91%)		

CPC: Cerebral Performance Category; IQR: Interquartile Range; CA: Cardiac Arrest; VF/VT: Ventricular Fibrillation/Ventricular Tachycardia; ROSC: Return Of Spontaneous Circulation; ICU: Intensive Care Unit; WLST: Withdrawal of Life-Sustaining Therapies

*mortality among the cohort (*N* = 155)

Compared to unfavorable outcome, patients were younger in the favorable outcome group (median age 66.5 yo IQR [54.8; 75] vs. 58 IQR[50; 65.5], *p* = 0.005). Time to ROSC (median 18 min IQR [11; 24] vs. 25 min IQR [21.8; 34], *p* < 0.001) and shockable initial rhythm (41 vs. 72%, *p* = 0.001) were significantly different between unfavorable and favorable outcome groups. Myocardial ischemia was the main cause of CA in the favorable outcome group, while respiratory failure was predominant in the unfavorable outcome group (*p* < 0.001).

Compared to included patients, non-included patients had a lower time to ROSC (median 20 min IQR [12; 28] vs. median 24.5 min IQR [20; 31.7], *p* = 0.002), a higher frequency of cardiac causes of CA (51 vs. 30%, *p* < 0.001), a lower rate of respiratory failure cause of CA (21 vs. 41%, *p* < 0.001) and a lower ICU mortality rate (41% vs. 71%, *p* < 0.001)(ESM4 - Table 1 supplementary material).

### EEG analysis

EEG recordings were obtained at a median time of 2[1; 3] days after CA, namely after the end of TTM. Benign, malignant and highly malignant patterns were observed in 54 (35%), 53 (34%) and 48 (31%) patients, respectively.

EEG patterns differed significantly between favorable and unfavorable outcome patients. In the favorable outcome group, EEG was benign in 87%, only 13% had a malignant pattern and none presented a highly malignant pattern. In the unfavorable outcome group, 17.2% had a benign pattern, 41.4% a malignant and 41.4% a highly malignant pattern (*p* < 0.001). 

About EEG background, a continuous or a nearly continuous background was also more frequently observed in the favorable outcome group (92 vs. 66%, *p* = 0.001). Conversely, burst-suppression pattern tends to be more frequently observed in the poor outcome group (13 vs. 0%, *p* = 0.18).

Regarding epileptiform features, none of patients with a favorable outcome presented a rhythmic or periodic patterns (RPPs) (0 vs. 27%, *p* < 0.001). Electrographic seizures or SE were only observed in 5% and 8% of favorable and unfavorable outcome patients, respectively (*p* = 0.50).

Regarding EEG reactivity, background was mostly reactive (95%) in the favorable outcome group, and mostly unreactive (66%) in the unfavorable outcome group (*p* < 0.001) (Table 2).

**Table 2:** Prognostic markers according to favorable and unfavorable neurological outcome at 3 months

	Good outcome CPC 1–2 *n* = 39	Poor outcome CPC 3-4-5 *n* = 116	*p*-value
**EEG patterns**			
Benign. n (%)	34 (87)	20 (17.2)	***< 0.001***
Malignant. n (%)	5 (13)	48 (41.4)	
Highly malignant. n (%)	0 (0)	48 (41.4)	
**EEG background**			
Continuous or nearly continuous. n (%)	36 (92)	76 (66)	***0.001***
Burst suppression. n (%)	0 (0)	15 (13)	*0.18*
**EEG epileptiform features**			
Rhythmic or periodic patterns (RPPs). n (%)	0 (0)	31 (27)	***< 0.001***
Seizure/status epilepticus. n (%)	2 (5)	9 (8)	*0.50*
**Reactive EEG** n (%)	37 (95)	39 (34)	***< 0.001***
NSE at 72 h* median [IQR]	20 [15; 30]	110 [49; 308]	***< 0.001***
**Bilaterally absent N20 SSEP** n (%)	0/11 (0)	32 /80 (40)	***0.009***
**Myoclonus** n (%)	7 (18)	62 (54)	***< 0.001***

CPC: Cerebral Performance Category; EEG: Electroencephalogram; NSE: Neuron Specific Enolase; SSEP: Somatosensory Evoked Potentials.

*According to the protocol, NSE at 48h was included when 72 hour-NSE was not available, accounting for 22 patients (14%)

### NSE, myoclonus and SSEP analyses

NSE level at 72 h was available in 133/155 patients (86%). Thus, we considered the NSE at 48 h in the other cases (22/155 patients, 14%). NSE levels at 72 h were significantly lower (median 20 µg/L IQR [15; 30]) in the favorable outcome compared to the unfavorable outcome group (median 110 µg/L IQR [49; 308], *p* < 0.001). Myoclonus was observed in 7 (18%) patients in the favorable outcome group and in 62 (54%) patients in the unfavorable outcome group (*p* < 0.001).

Finally, SSEPs were performed in 91 patients. N20 component was bilaterally absent in 32/80 patients (40%) in the unfavorable outcome group and was present in all patients with a favorable outcome (*p* = 0.009) (Table 2).

### NSE and EEG combined evaluation

We assessed the NSE level according to the three EEG patterns described by Westhall et al. (Fig 1.A). For favorable outcome patients, NSE was not significantly different among benign (median 19.6 µg/L IQR [15.6; 28.8]) and malignant EEG patterns (median 24.4 µg/L IQR [20; 27.8], *p* = 0.56). In patients with unfavorable outcome, NSE was significantly higher in highly malignant patterns (median 197 µg/L IQR [70; 429.8]) compared to benign EEG (median 62 µg/L IQR [37; 130.4], *p* = 0.003). The difference of NSE level between malignant and benign EEG did not reach significance (*p* = 0.18).

We also assessed the NSE level according to epileptic features (Fig. 1.B). Compared to all other EEG patterns, seizures or SE were not significantly associated with an increase in the NSE level (seizures or SE: median NSE level 67.7 µg/L IQR [34.6; 104]; other patterns: median 65.9 µg/L IQR [24.2; 275], *p* = 0.52). When comparing seizures or SE only to other malignant EEG patterns, no difference in NSE level was observed either (seizures or SE: median NSE level 50.8 µg/L IQR [29.6; 104] vs. other malignant patterns: median 164 µg/L IQR [49.8; 332], *p* = 0.15).

Finally, NSE level was assessed according to the EEG background (ESM5 - Fig. 2 supplementary material). In the favorable outcome, no difference in NSE level was observed between “normo-voltage and continuous background” and “discontinuous or low-voltage background” (p = 0.8). In the unfavorable outcome group, NSE values were significantly higher in case of “suppression or burst-suppression” compared to “continuous and normo-voltage background” (*p* < 0.001). The NSE level was also significantly higher in case of “discontinuous or low-voltage background” compared to “continuous and normo-voltage background” (*p* = 0.01). The difference of NSE level between “suppression or burst-suppression” and “discontinuous or low-voltage background” did not reach significance (*p* = 0.13).

### Prognostic value of NSE level according to EEG patterns

We assessed the prognostic value of NSE level according to EEG patterns (Table 3):

- When EEG was classified as benign, NSE was predictive of an unfavorable outcome with an area under the ROC (AUROC) curve of 0.80 CI95% (0.65–0.95). The recommended threshold of NSE > 60 µg/L predicted unfavorable outcome with a specificity of 94% and a sensitivity of 50%. A NSE > 78.2 µg/L was the optimal cut-off for unfavorable outcome prediction, permitting a specificity of 100% despite a sensitivity of 45%.
- When EEG was classified as malignant, NSE was predictive of a unfavorable outcome with a AUROC of 0.89 CI95% (0.78-1.0). A NSE > 60 µg/L predicted a poor outcome with 100% specificity despite a sensitivity of 66%. A NSE level > 45.2 µg/l enables 100% specificity with an optimized sensitivity of 70.8%.
- When seizures or SE were observed, NSE was predictive of a poor outcome with an AUROC of 0.83 CI95% (0.58-1.0). A NSE > 53.5 µg/L predicted unfavorable outcome with 100% specificity and a higher sensitivity (77%) compared to NSE > 60 µg/l (66%).
- Analysis with highly malignant patterns could not be performed as none of these patients had a favorable outcome.

**Table 3:**
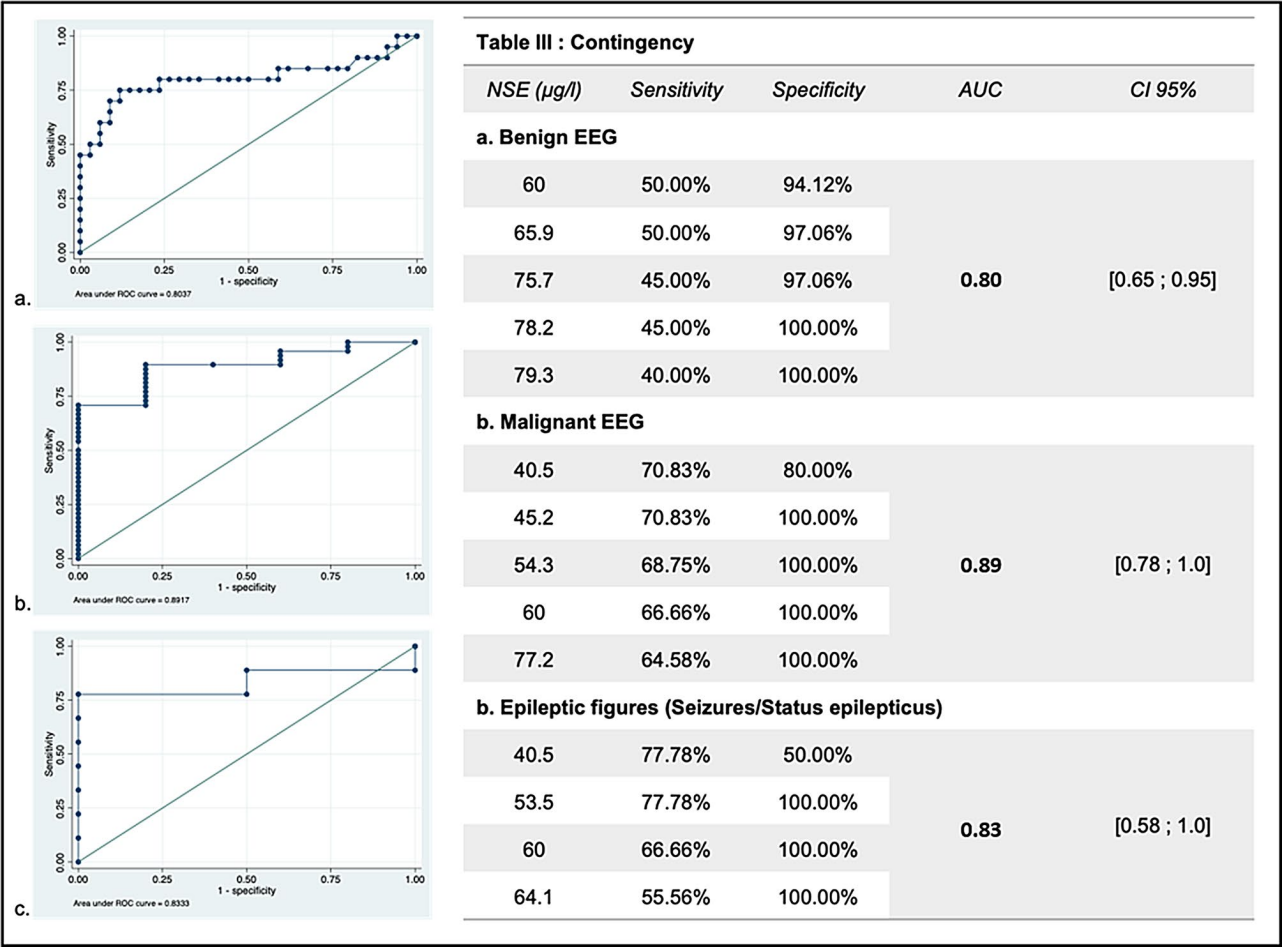
NSE prognostic value according to EEG patterns. (**a**). receiver operating curve (ROC) and corresponding NSE values when the EEG was benign. (**b**). ROC and NSE values when the EEG was malignant, without seizures or status epilepticus. (**c**). ROC and NSE values when EEG highlighted seizures or status epilepticus. EEG: electroencephalogram; AUC: area under the curve; CI: confidence interval; NSE: neuro-specific enolase

## Discussion

In this cohort of comatose patients after CA, we found a strong positive correlation between increasing EEG severity and increasing blood NSE level. We also highlighted that the recommended NSE level threshold of 60 µg/L at 48 and/or 72 h was not 100% specific of unfavorable outcome, in particular when the EEG pattern was benign (i.e., continuous and reactive, without malignant features). In this case, a NSE > 78.2 µg/L was 100% specific of unfavorable outcome, despite a limited sensitivity of 45%. We also highlighted that the best NSE threshold was > 45.2 µg/L in case of malignant EEG, and > 53.5 µg/L in case of seizure or SE. These thresholds were 100% specific of unfavorable outcome, with a higher sensitivity compared to the recommended threshold of 60 µg/L. Thus, our results suggest that the threshold of NSE could be personalized according to EEG pattern. Some recent studies already highlighted the significant positive correlation between the severity of EEG background and the NSE level [17, 18]. To our knowledge, this is the first study providing a modulated approach of NSE threshold based on the severity of brain injury reflected by EEG patterns.

Similarly to results of larger cohorts [7, 15], we highlighted that the AUC of NSE was below 90%, highlighting the limited prognostic performance of isolated NSE level and the necessity to use a multimodal approach. Each prognostic marker presents an imperfect sensitivity and specificity when used alone. Indeed, a highly malignant EEG can be observed in patients with an unfavorable outcome, although NSE value remain lower than 60 µg/L. A possible explanation would be that some patients could present severe HIBI in key areas for consciousness (basal ganglia and thalamic injuries), but not necessarily diffuse and extensive cortical lesions, explaining the limited NSE values [30]. Conversely, patients with benign EEG could present high NSE values and an unfavorable outcome, enhancing the limitation of EEG in some cases. The hypotheses to explain this could be the limited spatial resolution of EEG due to the limited number of sensor, the difficulties of EEG interpretation and the variation of EEG patterns through time [5, 31–33]. The kinetic of EEG pattern could also be an interesting prognostic marker, as some patterns could evolve from benign to malignant and vice versa [5, 31–33]. Thus, our combined approach of functional (EEG) and structural (NSE) markers of brain injury appears to be promising due to the individual limitations of each tool. Our study also suggest that assessment of HIBI using malignant EEG patterns remain challenging. Indeed, prognostic value of malignant EEG remains very heterogeneous among the different patterns, with a false positive rate between 0 and 33% according to studies [5, 7]. Our results are congruent with these data, as 13% of patients with a favorable outcome and 41% with an unfavorable outcome presented a malignant EEG. Thus, the prognostic performance of these malignant EEG patterns remains uncertain, highlighting the need to combine markers for poor outcome prediction.

We also examined the influence of electrographic seizures on NSE levels, which has never been done to date. Indeed, the pathophysiology of HIBI combines complex mechanisms related to brain hypoxia and ischemia, metabolic acidosis, neuroinflammation, mitochondrial dysfunction, and excitotoxicity [8]. Excitotoxicity could thus promote electrographic seizures. However, these seizures could also induce secondary brain damage [34]. Some studies conducted in non-CA status epilepticus cohorts highlighted that excitotoxicity and neuronal apoptosis induced by SE could be assessed by NSE level. Importantly, the median NSE level remains relatively low (between 18 and 20 µg/L) compared to those observed in HIBI [35]. Seizures in CA patients could thus increase the NSE level, the best threshold for poor outcome prediction being possibly higher than the recommended cut-off value. Our results suggested that NSE levels did not differ between patients with electrographic seizures and patients with a supposed relatively similar degree of brain injury, i.e. others malignant EEG patterns (RPPs, discontinuous, low voltage and unreactive EEG). Consequently, SE should not be considered as a confounding factor of NSE level. These results are relatively congruent with the recent study of Tziakouri et al., that analyzed the variability of NSE distribution according to epileptiform discharges. During late EEG recording (i.e. 36 to 72 h after AC), the authors found no difference in NSE levels between discontinuous background with epileptiform discharges and discontinuous background without epileptiform discharge [18]. Briefly, our results suggest that secondary neuronal injuries due to epileptic features could be limited, and that electrographic seizures could be considered as a marker of HIBI more than a severe secondary brain insult. Nevertheless, our analysis was limited to a quantitative measure of neuronal injury using NSE, and we did not assess the regional cerebral blood flow or brain tissue oxygenation using invasive brain monitoring [34]. To assess whether SE is a cause or a consequence of brain damage after CA, we could evaluate the interest of treating these patterns with antiseizures medications (ASMs). However, studies regarding benefice of ASMs in this situation remain discordant. Some cohort studies highlighted a favorable outcome between 6 and 44% if treated with aggressive ASMs [12, 13]. Conversely, Ruijter et al. recently demonstrated that systematic ASMs of epileptic and epileptiform features did not improve the neurological prognosis [11]. This randomized trial included SE but also epileptiform features which do not systematically require ASMs. Thus, it is difficult at this point to ascertain the interest or futility of ASMs, although the majority of patients presented an unfavorable outcome in most of studies [5, 7, 11, 12]. Overall, our results show that a NSE > 53 µg/l was 100% specific of unfavorable outcome in case of electrographic seizures, suggesting that there is no reason to increase the NSE cut-off compared to patients without epileptic features.

Regarding the strengths of our study, we included patients still comatose after CA, in concordance with current guidelines for neuroprognostication [4, 36]. EEG recording is standardized in our center, the interpretation was blinded to other prognostic tools and performed by two expert neurophysiologists. We assessed neurological outcome at 3 months, permitting a long-term outcome assessment. We also assessed some other prognostic markers, i.e., myoclonus and SSEP.

The main limitation of our study is the monocentric setting with a limited sample size, possibly related to the selection of patients that were still comatose after CA. Indeed, early mortality related to refractory shock in the first 48 h after CA is significant [2]. However, this population was selected for its clinical relevance to the question of neuroprognostication. Secondly, the physician in charge was not blinded to the results of EEG and NSE. Consequently, we cannot exclude that this may have affected patients’ management regarding WLST and self-fulfilling prophecy, a common bias in this setting. Nevertheless, the WLST decisions did not included the threshold of 60 µg/l, as our study was mainly conducted before the 2021 guidelines that described this specific threshold [29, 36]. Furthermore, we only used highly malignant EEG in our prognostication algorithm, limiting the risk of WLST for patients with a malignant or a benign EEG pattern. As recommended by current guidelines, we also used a multimodal approach for poor outcome prediction, limiting the risk of unjustified WLST. We did not assess the correlation between EEG and other biomarkers of neuronal injury, as neurofilament light chain (NFL) [37]. Although this biomarker seems to be highly predictive of unfavorable outcome (AUC between 0.92 and 0.97), NFL accessibility is limited in routine practice [38–40]. We did not report data regarding brain imaging (CT-scan or MRI). Indeed, our most common practice is to perform brain imaging upon admission, in order to eliminate a primary neurological cause of CA, and we recognize that anoxo-ischemic lesions may be underestimated at this stage [41]. We lack data on exact timing between sedation weaning and EEG recording. Nevertheless, EEG recordings were performed according to ESICM/ERC recommendations, i.e., in case of persistent coma after sedation weaning. Thus, we included only patients that presented a GCS < 8 at admission and excluded early awaking after CA. Moreover, EEG were performed at a median time of 2[1; 3] days, i.e., after the end of TTM that usually require sedation.

Our result highlighted that discrepancy between prognostic markers remains a red-flag and reflects complex pathophysiological mechanisms. Thus, adjustment of NSE thresholds according to EEG patterns could be of importance in this decision-making process. Larger prospective studies are required to confirm these results.

## Conclusion

In comatose patients after CA, we found a positive correlation between increasing EEG severity and increasing NSE level, emphasizing the concordance between functional and structural markers of HIBI. Our results also suggest that modulating the NSE threshold according to the EEG pattern could optimize the prognostic performance to predict unfavorable outcome. Furthermore, the NSE level did not differ between electrographic seizures and other malignant EEG patterns, suggesting that seizures could be a marker of HIBI severity more than a secondary brain insult after CA.

## Electronic supplementary material

Below is the link to the electronic supplementary material.

Supplementary Material 1

## Data Availability

Data could be available if request to corresponding author, to a reasonable extent.

## Abbreviations

AUROC: Area Under the ROC
CA: Cardiac Arrest
CPC: Cerebral Performance Category
EEG: Electroencephalogram
ESM: Electronic Supplementary Material
GCS: Glasgow Coma Scale
HIBI: Hypoxic Ischemic Brain Injury
ICU: Intensive-Care-Unit
IQR: Interquartile-Range
NSE: Neuron-Specific Enolase
RASS: Richmond-Agitation-Sedation-Scale
ROSC: Return of Spontaneous Circulation
SD: Standard Deviation
SE: Status Epilepticus
SSEP: SomatoSensory Evoked Potentials
TTM: Targeted Temperature Management
WLST: Withdrawal-of-Life-Sustaining-Therapies
µV: MicroVolt
